# Development of a Glucose Sensor Based on Glucose Dehydrogenase Using Polydopamine-Functionalized Nanotubes

**DOI:** 10.3390/membranes11060384

**Published:** 2021-05-24

**Authors:** Won-Yong Jeon, Hyug-Han Kim, Young-Bong Choi

**Affiliations:** 1 School of Chemical Engineering and Biomedical Institute for Convergence at SKKU (BICS), Sungkyunkwan University, Suwon 16419, Korea; powerwy@skku.edu; 2 Department of Chemistry, College of Science & Technology, Dankook University, Dandae-ro, Cheonan-si 31116, Chungnam, Korea; hankim@dankook.ac.kr

**Keywords:** glucose dehydrogenase, electrochemical biosensor, PDA-MWCNT, redox mediator

## Abstract

The electrochemical-based detection of glucose is widely used for diagnostic purposes and is mediated by enzyme-mediated signal transduction mechanisms. For such applications, recent attention has focused on utilizing the oxygen-insensitive glucose dehydrogenase (GDH) enzyme in place of the glucose oxidase (GOx) enzyme, which is sensitive to oxygen levels. Currently used Ru-based redox mediators mainly work with GOx, while Ru(dmo–bpy)_2_Cl_2_ has been proposed as a promising mediator that works with GDH. However, there remains an outstanding need to improve Ru(dmo–bpy)_2_Cl_2_ attachment to electrode surfaces. Herein, we report the use of polydopamine-functionalized multi-walled carbon nanotubes (PDA-MWCNTs) to effectively attach Ru(dmo–bpy)_2_Cl_2_ and GDH onto screen-printed carbon electrodes (SPCEs) without requiring a cross–linker. PDA-MWCNTs were characterized by Fourier transform infrared (FT–IR) spectroscopy, Raman spectroscopy, and thermal gravimetric analysis (TGA), while the fabrication and optimization of Ru(dmo–bpy)_2_Cl_2_/PDA-MWCNT/SPCEs were characterized by cyclic voltammetry (CV) and electrochemical impedance spectroscopy (EIS) measurements. The experimental results demonstrate a wide linear range of glucose-concentration-dependent responses and the multi-potential step (MPS) technique facilitated the selective detection of glucose in the presence of physiologically relevant interfering species, as well as in biological fluids (e.g., serum). The ease of device fabrication and high detection performance demonstrate a viable pathway to develop glucose sensors based on the GDH enzyme and Ru(dmo–bpy)_2_Cl_2_ redox mediator and the sensing strategy is potentially extendable to other bioanalytes as well.

## 1. Introduction

Glucose is a major energy source for cellular activity in vivo, and maintaining an appropriate physiological concentration of glucose is important for human health [1]. Owing to the inability to control glucose levels within the appropriate range, diabetes is one of the leading causes of death and disability, and the diagnosis and management of diabetes requires the strict monitoring of glucose levels [2]. The normal glucose level in healthy humans is between 75 to 125 mg/dL in the absence of eating. Diabetes is determined when the glucose level is above 126 mg/dL in blood without having had a meal for 8 h. It can also be judged when the glucose level is above 200 mg/dL in blood 2 h after a meal [3]. Among different monitoring options, the electrochemical biosensor is widely used for glucose monitoring, and the electrochemical glucose sensor technology utilizes the glucose oxidase (GOx) enzyme and an electron transfer mediator such as p–benzoquinone, phenazine ethosulfate, lutetium phthalocyanine, or osmium [4,5,6,7,8,9]. Commercial blood glucose monitoring devices on the market mainly utilize a second-generation glucose sensor that consists of a metal mediator and GOx enzyme [10]. Within second-generation glucose sensors, glucose is oxidized by GOx to produce gluconic acid and H_2_O_2_ [6,11], and electrons generated by glucose oxidation are transferred to the electrode via an electron transfer mediator such as iron or ruthenium—a sensing concept which has been employed in commercial glucose biosensors [9,10,12]. GOx has a high temperature and pH stability along with excellent glucose substrate selectivity [13,14,15]; however, GOx uses O_2_ as an external electron acceptor in the oxidation reaction so device performance is sensitive to and variable depending on the atmospheric oxygen level [16,17,18]. To overcome this challenge and improve sensing reliability, glucose dehydrogenase (GDH) does not require O_2_ and is hence being used in various types of glucose sensors together with pyrroloquinoline (PQQ), nicotinamide adenine dinucleotide (NAD), and flavin adenine dinucleotide (FAD) redox cofactors [19,20,21]. Among them, FAD–GDH has excellent thermal stability and substrate selectivity and does not require additional cofactors or active catalysts [21,22,23]. However, FAD–GDH has ineffective electron transfer with the commercial Ru(NH_3_)_6_ mediator that is used with GOx, and Ru(dmo-bpy)_2_Cl_2_ has been reported to be an effective redox mediator for electron transfer with FAD-GDH [24]. The effective attachment of enzymes and redox mediators to electrode surfaces remains an important, yet challenging aspect of sensor design, especially for Ru(dmo-bpy)_2_Cl_2_, and various physical and chemical methods, such as adsorption, entrapment, cross-linking, and covalent coupling are being explored [25].

However, currently used attachment methods have several drawbacks as follows: (1) adsorbed enzyme is only on a specific part of the electrode surface, resulting in poor reproducibility; (2) the enzyme cannot be firmly bonded to the electrode and readily desorbs; (3) the performance of the glucose sensor is reduced by a diffusion barrier, and (4) surface attachment may cause detrimental changes in enzyme conformation and hence enzymatic activity [26,27]. As one promising solution, polydopamine (PDA) coatings are attractive because they are biocompatible and have many types of functional groups such as catechols, amines, and imines to facilitate high-density enzyme attachment [28]. The excellent adhesion properties of PDA have been reported on various surfaces such as MWCNTs, graphene, magnetic nanoparticles, and glassy carbon electrodes [29,30,31,32]. Among the different options, PDA-MWCNTs have demonstrated particularly high sensitivity levels and limits of detection for electrochemical biosensors [33].

**Scheme 1:**
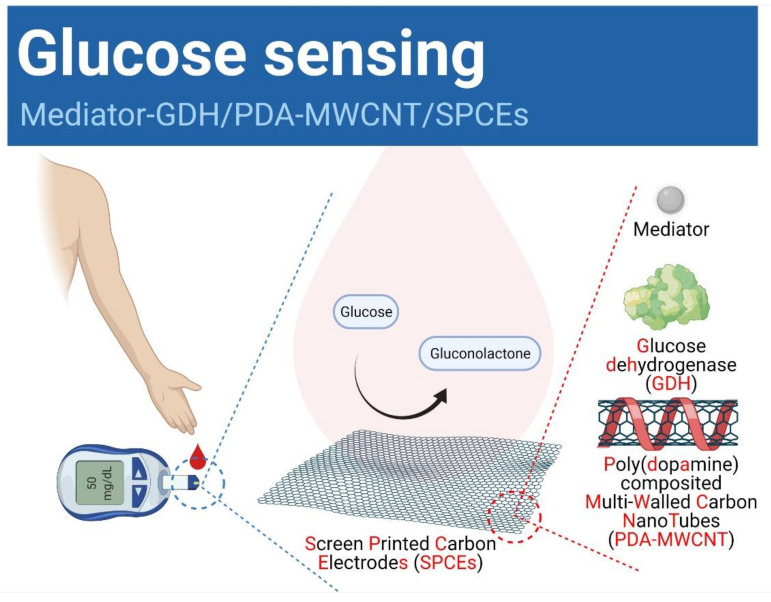
Concept and composition concept of glucose sensing system.

In this study, PDA-MWCNT was synthesized simply by changing the conditions of ultra-sonication using PDA and MWCNT in an alkaline environment. The synthesized PDA–MWCNT was fixed at room temperature on screen-printed carbon electrodes (SPCEs), and the GDH enzyme and Ru(dmo–bpy)_2_Cl_2_ mediator which was synthesized as described in the previous study [24] were cast (Scheme 1). The GDH enzyme and Ru(dmo–bpy)_2_Cl_2_ mediator could be readily immobilized without a cross-linker due to the amine and catechol groups on the PDA-MWCNT-modified SPCE. In addition, the Ru(dmo–bpy)_2_Cl_2_/GDH/PDA–MWCNT/SPCE exhibits the selective detection of glucose by the GDH enzyme, even in the presence of interfering species. On the electrode adsorbed with PDA-MWCNT, the current signal for the oxidation catalyst of glucose was five times higher than that of general SPCEs. Thus, it was confirmed that the sensitivity of the glucose sensor and the linearity of the concentration sensitivity were increased. It has been shown that it can be applied well as a new second generation glucose sensor.

## 2. Materials and Methods

### 2.1. Chemicals and Reagents

To fabricate PDA-MWCNT, dopamine hydrochloride and tris(hydroxymethyl)aminomethane were purchased from Sigma–Aldrich Co. (Milwaukee, Brookfield, WI, USA), and model MR99 (MWCNT; >99 wt%, 5–15 nm diameter, ~20 µm length) was purchased from Nano-material Technology Co. (Pohang, Korea). To measure the electrochemical impedance spectroscopy (EIS), potassium hexacyano ferrate(Ⅱ) trihydrate and potassium hexacyanoferrate(Ⅲ) (Sigma–Aldrich Co.) were used. Human serum samples were purchased from Sigma–Aldrich (S1-M). Analytical reagents such as D–(+)–glucose, uric acid, ascorbic acid (Sigma–Aldrich Co.) were used without further purification. Glucose dehydrogenase (FAD-dependent GDH-584 U/mg) was purchased from Totobo Co. (Osaka, Japan). The carbon paste (Electrodag 423SS, Acheson, Irvine, CA, USA) as a working electrode was screen-printed on an overhead projector (OHP film using a screen-printing machine (BS–860AP, Bando, Korea). The mediator, Ru(dmo–bpy)_2_Cl_2_, was synthesized as previously described [24]. Phosphate-buffered saline (1X PBS, 4.3 mM NaH_2_PO_4_, 15.1 mM Na_2_HPO_4_, and 140 mM NaCl) and all other solutions were prepared using deionized Milli-Q water (Millipore, Bedford, MA, USA; Registered 18 M·Ωcm at 25 °C).

### 2.2. Fabrication of Ru(dmo-bpy)_2_Cl_2_/GDH/PDA-MWCNT/SPCEs

#### 2.2.1. Synthesis of PDA-MWCNT

The 10 mM tris buffer of pH 8.8 was prepared by adding tris(hydroxymethyl)aminomethane (72.615 mg) in 60 mL of DI water. The different amounts of dopamine hydrochloride (120, 150, 180, 210, 240, and 270 mg) were added and reacted in the as-prepared 10 mM tris buffer for 24 h. Then, the PDA color turned dark brown. To check the synthesis of PDA, FT–IR was carried out. To make the PDA-MWCNT composite, 30 mg of MWCNT was added and sonicated for 1 h, followed by incubation at room temperature for 24 h. Then, PDA-MWCNT could be dispersed well in DI water. Next, the PDA-MWCNT composite was filtered using a 0.2 μm diameter nylon membrane filter with DI water three times. Finally, the PDA-MWCNT composite was dispersed in 30 mL of DI water. The composite of PDA-MWCNT was characterized by Raman and TGA (Rigaku, Japan), and morphological properties were characterized by high resolution–transmission electrons microscope (HR–TEM).

#### 2.2.2. Fabrication of Ru(dmo–bpy)_2_Cl_2_/GDH/PDA-MWCNT/SPCE

The different amounts of PDA-MWCNT solution were centrifuged to separate non-dispersed PDA-MWCNTs. The concentrations of the dispersed and centrifuged PDA-MWCNTs were 20.0 mg/mL and 15.0 mg/mL, respectively (Figure S1). We used the supernatant solution for our experiments. To fabricate the PDA-MWCNT-modified electrode, 10 μL of the supernatant solution was used for casting on the SPCEs and dried at room temperature for 24 h. Then, 10 μL of mixed solution (1:1 v/v) of GDH (40 mg/mL in 1X PBS) and Ru(dmo–bpy)_2_Cl_2_ (1 mg/mL in DI water) were cast and dried for 24 h on the PDA-MWCNT-absorbed SPCEs (Ru(dmo-bpy)_2_Cl_2_/GDH/PDA-MWCNT/SPCE). For electrochemical measurements, 5.5 mM glucose of 40 μL was loaded onto the Ru(dmo-bpy)_2_Cl_2_/GDH/PDA-MWCNT/SPCE. Then, cyclic voltammetry (CV) and multi-potential step (MPS) techniques were carried out at voltages ranging from –0.8 to 0.8 V with a scan rate of 20 mV/s.

### 2.3. Electrochemical Measurements

A CHI660B potentiostat (CH Instrument Inc. Austin, TX, USA) was used for all electrochemical experiments in which 0.5 mm Pt wire was a counter, Ag/AgCl (3.0 KCl Cypress, Lawrence, KS, USA) a reference, and 3.5 mm diameter SPCEs a working electrode (Figure S2). The EIS technique was used for the electrochemical characterization of the solid–liquid interface of SPCEs, PDA-MWCNT/SPCEs, and Ru(dmo-bpy)_2_Cl_2_/GDH/PDA-MWCNT/SPCEs. A mixed solution (40 μL) of 2.0 mM potassium hexacyanoferrate (II) trihydrate (in 0.5 M KCl) and 2 mM potassium hexacyanoferrate (III) (in 0.5 M KCl) for EIS measurements at the set frequency range from 10^5^ to 10^−3^ Hz, the AC amplitude of 10 mV at below 1 Hz, and DC potential at 0.286V. For the glucose determination, the CV and MPS experiments were performed in 1X PBS-based solution. In the MPS measurements, the initial potential was set at 0 V for 0.2 s and subsequent measurements were made every 0.1 V for 5 s from 0.1 to 0.5 V. For LOD, a blank sample was measured 10 times to calculate an average value and a standard deviation. Three standard substances of low concentration (0.1, 0.5, 1.0 mM) were prepared, and the background was corrected with the average blank value. The calibration curve was obtained with y = ax, where is the slope and x and y are the coordinates. After multiplying the standard deviation calculated by the blank sample by 3.3, it was divided by the slope (a) of the calibration curve. To check the effects of interferences, three physiologically relevant interfering species, 0.1 mM dopamine (DA), 0.1 mM ascorbic acid (AA), and 0.1 mM uric acid (UA), were carried out by CV. Finally, CV and MPS were carried out for checking real sample, glucose-spiked serum, by CV and MPS in Ru(dmo–bpy)_2_Cl_2_/GDH/PDA-MWCNT/SPCEs.

## 3. Results and Discussions

### 3.1. Physicochemical Characterization of PDA-MWCNT

**Figure 1:**
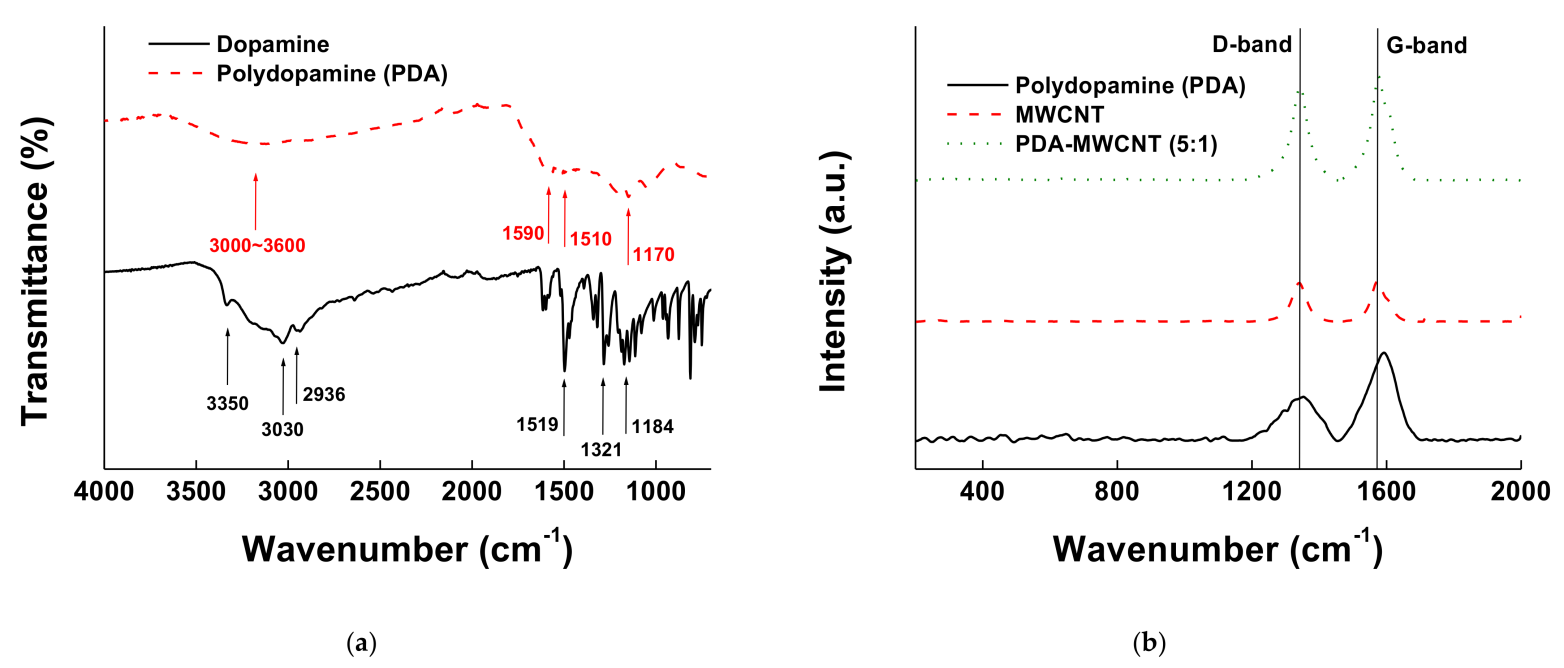

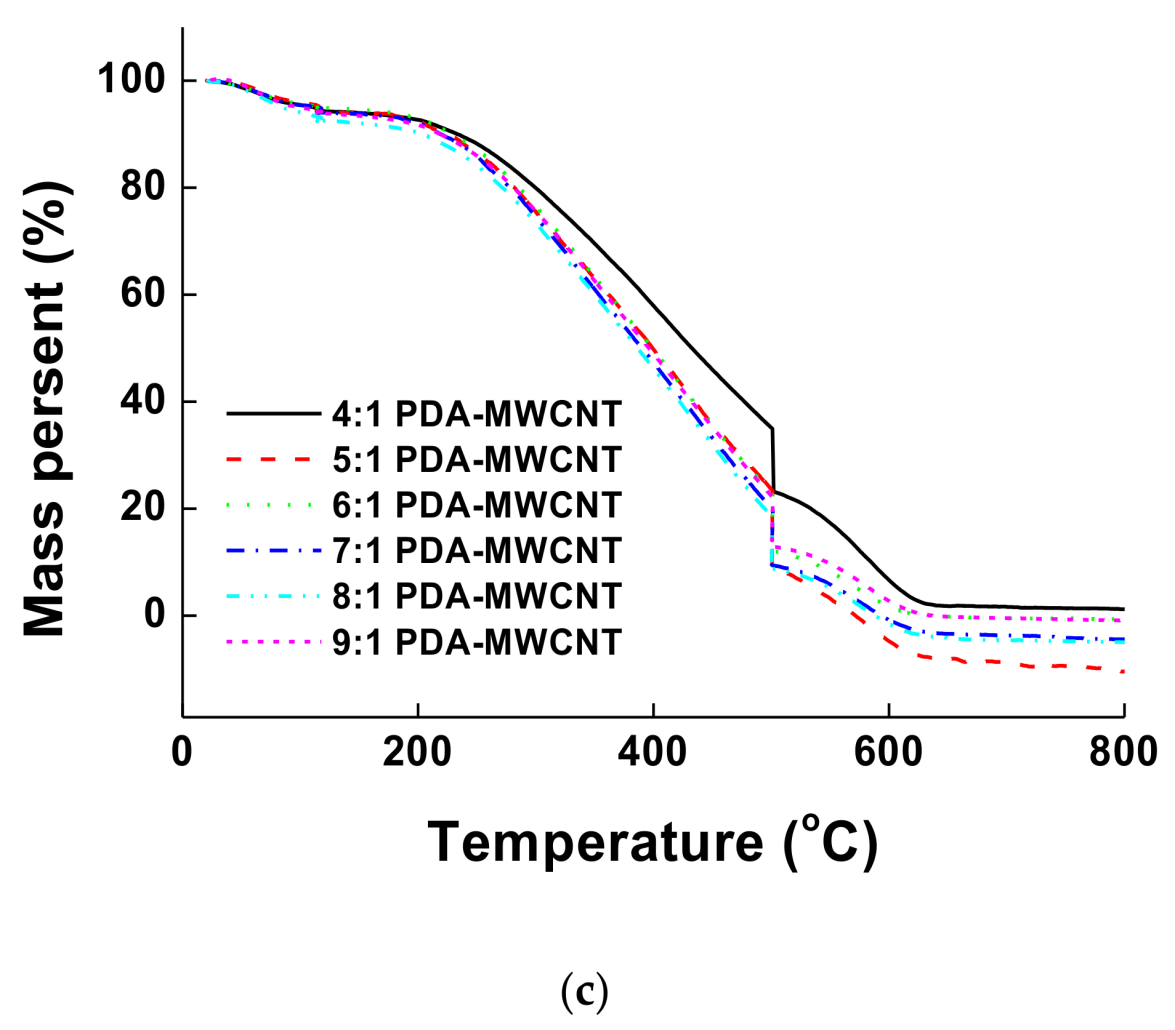
Characterization of PDA and PDA-MWCNT by (**a**) FT-IR, (**b**) Raman, and (**c**) TGA.

Firstly, the synthesized PDA was collected as a powder and its functionalization was characterized by FT–IR (Figure 1a). The dopamine (black line) showed the aromatic O–H stretching vibrations that were observed at 3030 cm^−1^ and 2936 cm^−1^, and the N–H (primary amine) stretching vibration and scissoring vibration were measured at 3350 cm^−1^ and 1519 cm^−1^, respectively. Additionally, the C–O–H bending vibration and C–O symmetry stretching vibration peaks were observed in 1321 cm^−1^ and 1184 cm^−1^, respectively [34]. The PDA (red line) showed the traditional broad stretching band of N–H (secondary amine) and O–H from 3000 to 3600 cm^−1^. Additionally, the aromatic C = C stretching peak of indole was shown at 1590 cm^−1^ and 1510 cm^−1^, and the C–N bending peak of the indolequinone at 1170 cm^−1^ [35]. Therefore, PDA was synthesized successfully by confirmation of the indolequinone structure.

Secondly, PDA-MWCNT composites were confirmed by the Raman spectrum in Figure 1b. Normally, traditional D and G bands of MWCNT (red line) at 1351 cm^−1^ and 1583 cm^−1^, respectively, indicate the preserved graphitic structure. Additionally, PDA-MWCNT composites (green line) synthesized by ultra-sonication for 1 h were measured. We calculated the *I_D_*/*I_G_* intensity ratio of PDA-MWCNT. Usually, the *I_D_*/*I_G_* intensity ratio shows the microstructural quality of MWCNT. A high *I_D_*/*I_G_* intensity ratio means defects of MWCNT [36]. The *I_D_*/*I_G_* intensity ratio of our PDA-MWCNT decreased to 0.876. That is the reason that the stretching and deformation of the PDA’s aromatic structure (black line) in 1580 cm^−1^ and 1350 cm^−1^ caused the increased G band of MWCNT. We evaluated non-defects of MWCNT by ultra-sonication, and the results showed good conformation of PDA-MWCNT composite.

Thirdly, the amount of PDA in samples which were prepared at various ratio of PDA to MWCNT (4, 5, 6, 7, 8, and 9:1) was observed by TGA between 25 and 800 °C in an air environment (Figure 1c). Normally, TGA shows the decreasing weight of the sample from the initial temperature. In Figure 1c, the moisture in the sample was removed by evaporation at around 100 °C, and the weight of PDA and MWCNT decreased from 200 °C and 500 °C, respectively. Additionally, the slopes are showing linearity because PDA was wrapped onto the MWCNT successfully.

**Figure 2:**
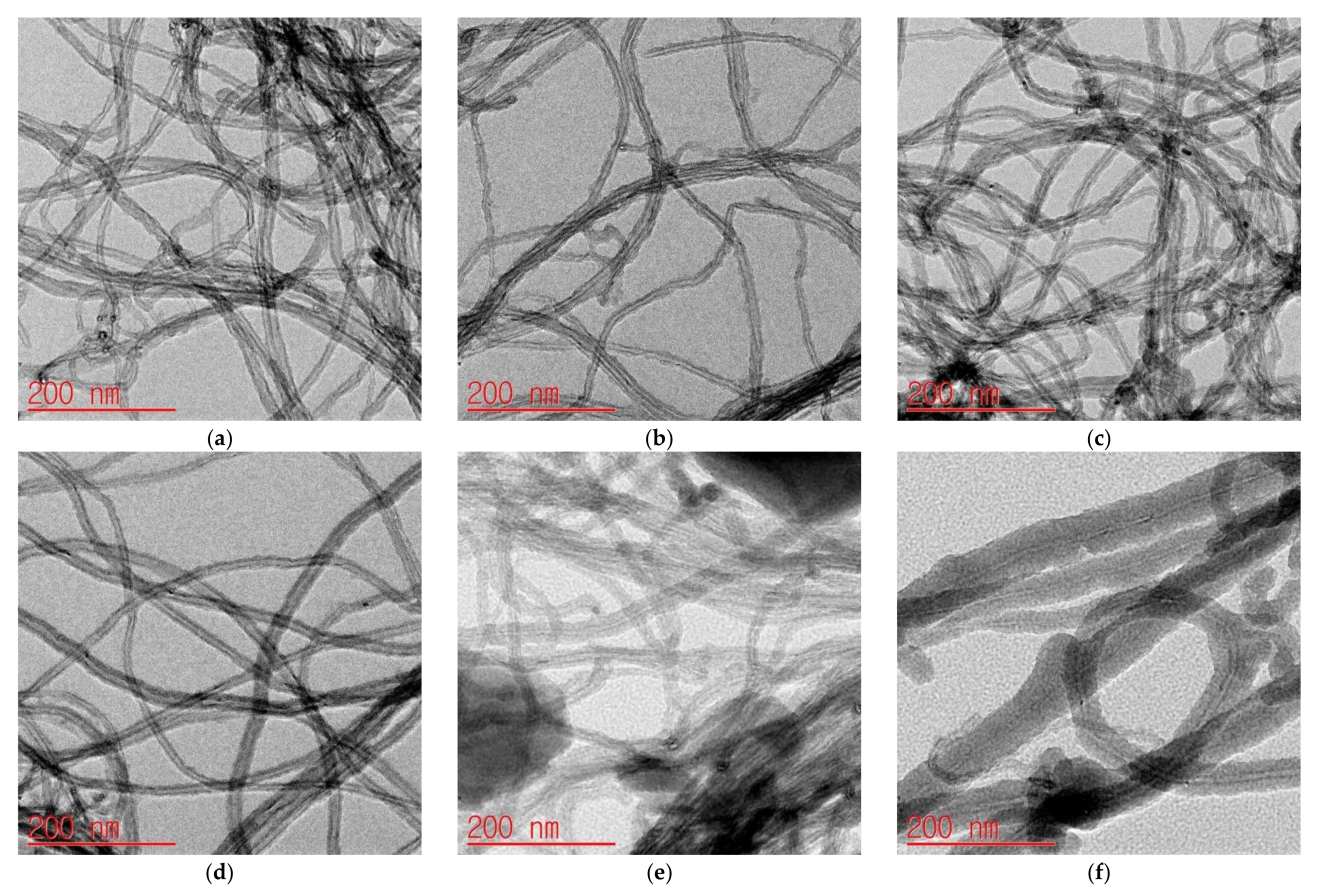
HR-TEM images of various ratio between PDA and MWCNT with (**a**) 4:1, (**b**) 5:1, (**c**) 6:1, (**d**) 7:1, (**e**) 8:1, and (**f**) 9:1.

Finally, the PDA-MWCNT composites were characterized by HR–TEM (Figure 2a–f), and the PDA-MWCNT ratios were 4:1, 5:1, 6:1, 7:1, 8:1, and 9:1. The starting MWCNTs are also presented in Figure S3. The results show that the thickness increased as the PDA ratio increased, which agrees with the results of the ratio between PDA and MWCNT determined by TGA. Table 1 indicates the real ratio of PDA to MWCNT mass and average thickness of PDA coated on the MWCNT surface.

### 3.2. Electrochecmial Characterization of Ru(dmo–bpy)_2_Cl_2_/GDH/PDA-MWCNT/SPCEs

**Figure 3:**
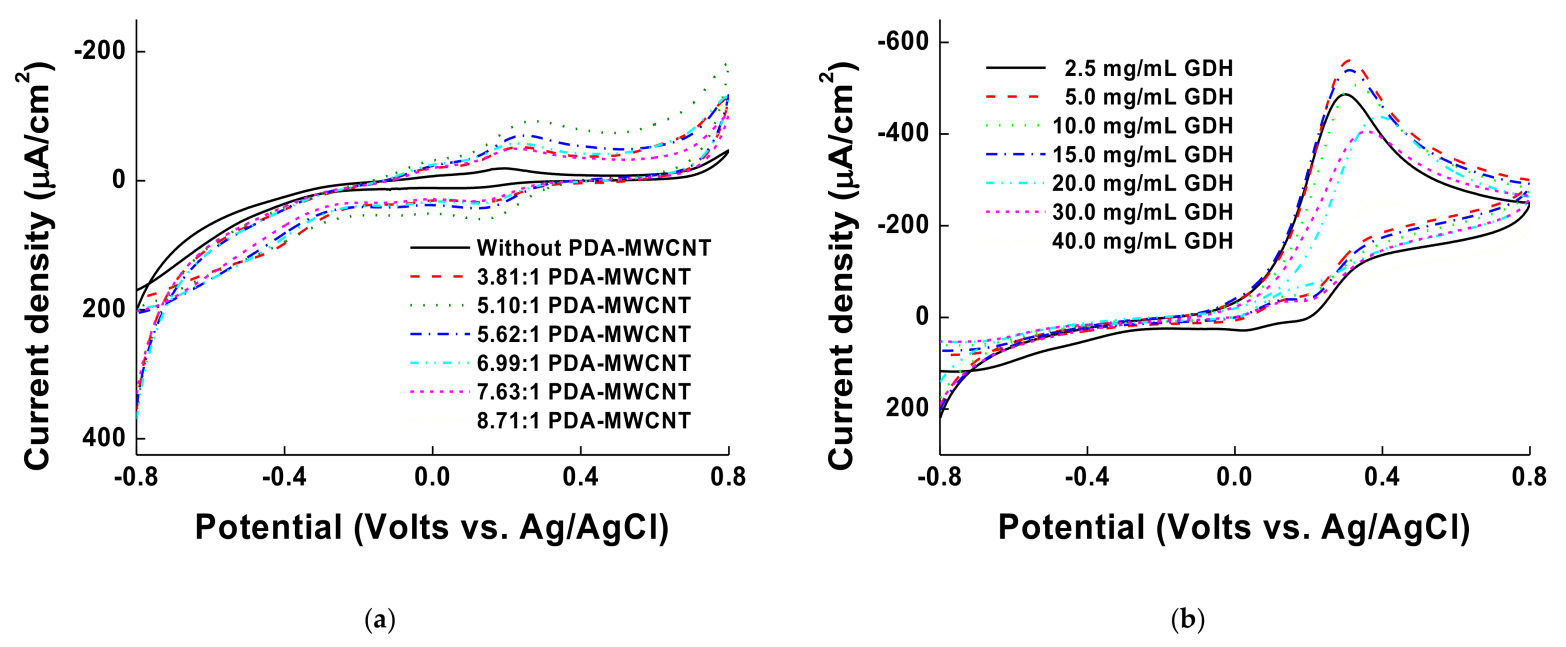
Cyclic voltammograms for optimization of (**a**) PDA-MWCNT ratio and (**b**) GDH amounts onto the electrode.

The electrochemical properties were evaluated by CV experiments (Figure 3). The 10 µL of PDA-MWCNT with various ratio was cast onto the SPCEs and dried for 24 h in desiccator. Then, 40 μL of Ru(dmo–bpy)_2_Cl_2_ (1.0 mg/mL in DI water: 1.6544 mM) was cast onto the PDA-MWCNT/SPCEs for measurements, and CV measurements were carried out by using 1X PBS (pH 7.0) at a scan rate of 20 mV/s (Figure 3a). The synthesized Ru(dmo–bpy)_2_Cl_2_ was observed at E° = 0.218 V in bare SPCEs. Ru(dmo–bpy)_2_Cl_2_ peaks in all ratios of PDA-MWCNT (3.81, 5.10, 5.62, 6.99, 7.63, and 8.71:1)-modified SPCEs increased, as compared to bare SPCEs due to increasing conductivity. Interestingly, the ratio of PDA-MWCNT (5.10:1) showed maximum redox peaks. We guess that the high London force of the PDA-MWCNT (5.10:1) ratio was affected. On the other hand, the redox peaks with ratios of PDA-MWCNT (5.62, 6.99, 7.63 and 8.71:1) decreased due to the increasing thickness of PDA [36]. It means that the amount of MWCNT has been decreasing when PDA thickness has been increasing. Therefore, the ratio of PDA-MWCNT (5.10:1) was optimized and selected to apply for the sensor. To optimize the concentration of the enzyme, various GDH concentrations (2.5, 5.0, 10.0, 15.0, 20.0, 30.0, and 40.0 mg/mL in 1X PBS) were mixed with Ru(dmo–bpy)_2_Cl_2_ (1 mg/mL in DI water). Then, 10 µL of mixed solution (GDH:Ru(dmo–bpy)_2_Cl_2_ = 1:1) was cast onto the PDA-MWCNT(5.10:1)/SPCEs, and dried for 24 h in a desiccator. Next, 5.5 mM glucose solution (in 1X PBS; 40 µL) was measured by CV at a scan rate of 20 mV/s (Figure 3b). The oxidation peak started from 0.0794V due to the absorbed Ru(dmo–bpy)_2_Cl_2_. Additionally, the maximum oxidation catalytic current was around 0.3 V within 5.0 mg/mL GDH condition. At higher GDH concentrations, the oxidation catalytic currents decreased continuously due to the maximized modification amount of 10.0, and 15.0 mg/mL GDH. In addition, we observed the solution aggregation at 20.0, 30.0, and 40.0 mg/mL GDH. Therefore, 5.0 mg/mL GDH was optimized and modified for the application of sensors.

**Figure 4:**
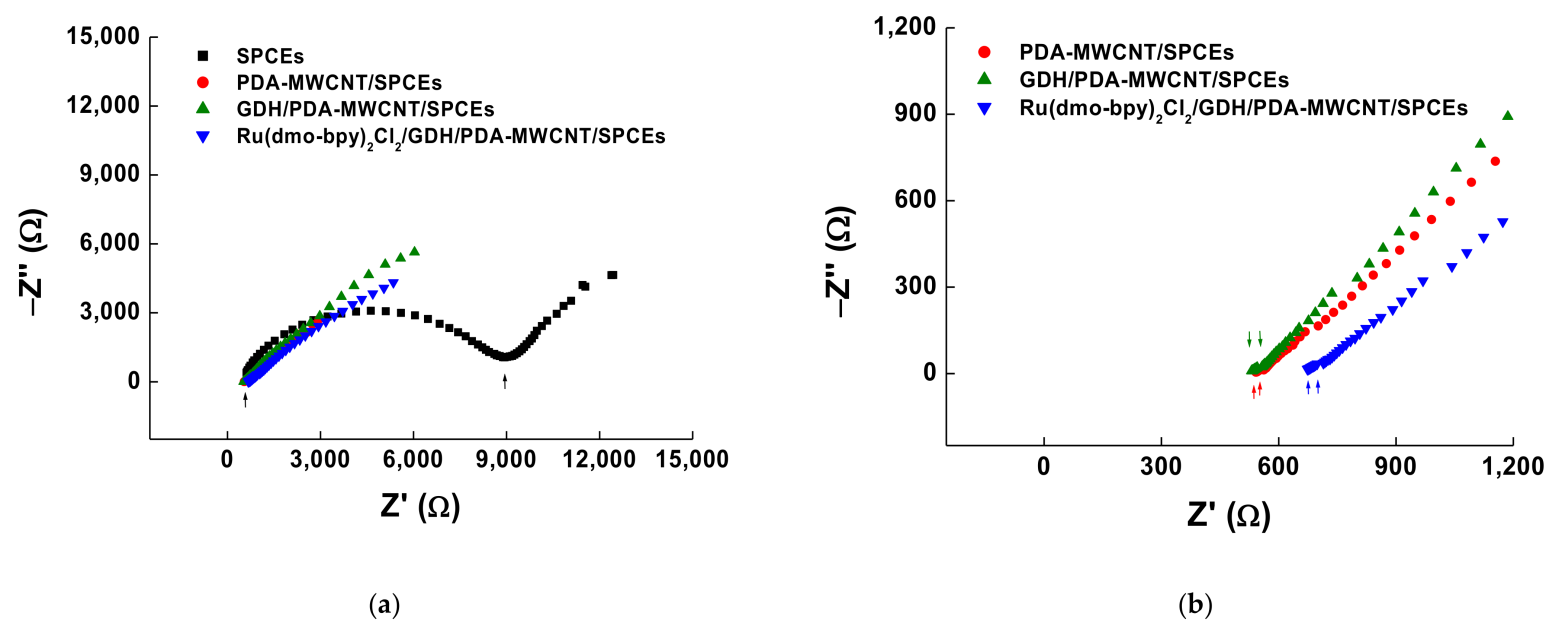
Nyquist plot of (**a**) normal and (**b**) zoom for bare SPCEs (black square), PDA-MWCNT/SPCEs (red circle), GDH/PDA-MWCNT/SPCEs (green triangle), and Ru(dmo-bpy)_2_Cl_2_/GDH/PDA-MWCNT/SPCEs (blue reverse triangle).

In the EIS results (Figure 4), semicircle diameters show the interface properties and electron transfer resistance (R_et_) of the electrodes [37]. Bare SPCEs (black square) had a resistance around 8808 Ω, while the resistance of PDA-MWCNT/SPCEs (red circle) decreased by 3 Ω (Figure 4a). That is the reason why the electron transfer catalytic effect of the conductive MWCNT caused the decrease in resistance. GDH/PDA-MWCNT/SPCEs (green triangle) and Ru(dmo-bpy)_2_Cl_2_/GDH/PDA-MWCNT/SPCEs (blue reverse triangle) had a resistance of 25 Ω and 39 Ω, respectively (Figure 4b). The increased resistance of GDH/PDA-MWCNT/SPCEs and Ru(dmo–bpy)_2_Cl_2_/GDH/PDA-MWCNT/SPCEs caused a slight decrease in electron transfer between liquid to MWCNT due to the protein and mediator. These results showed the effective modification of each material for electrode. Finally, we confirmed the interaction of the materials on the surface under each condition.

### 3.3. Glucose Measurement on Ru(dmo-bpy)_2_Cl_2_/GDH/PDA-MWCNT/SPCEs

**Scheme 2:**
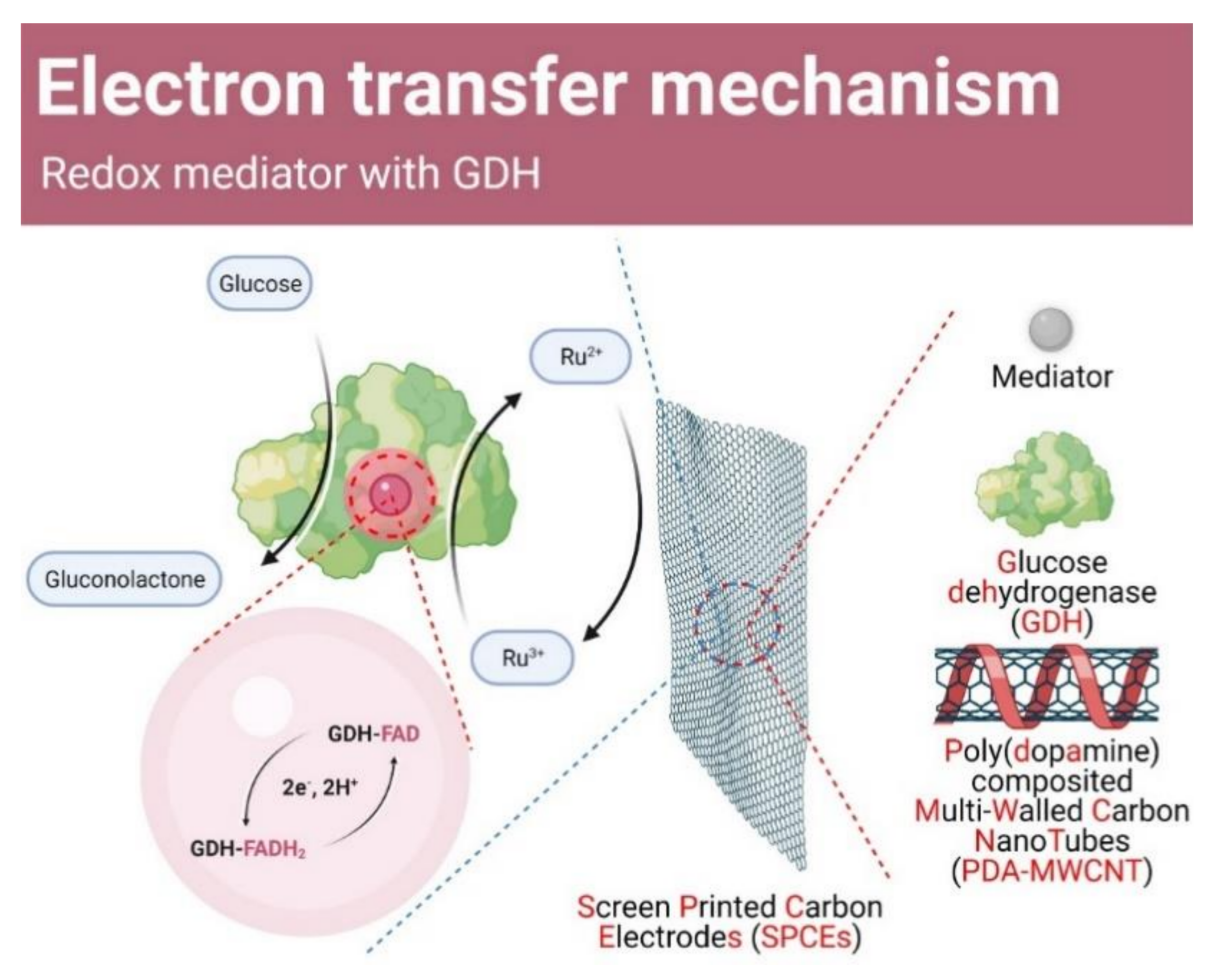
Electrochemical mechanism of GDH with glucose on Ru(dmo-bpy)_2_Cl_2_/GDH/PDA- MWCNT/SPCEs.

We measured different glucose concentrations of 0.0, 0.1, 0.5, 1.0, 5.0, 10.0, 15.0, 20.0, and 30.0 mM by CV and MPS for the Ru(dmo-bpy)_2_Cl_2_/GDH/PDA-MWCNT/SPCEs in 1X PBS (pH 7.0). The measurements were carried out in atmosphere conditions because GDH is not reactive in oxygen. Scheme 2 outlines the proposed mechanism of the electron transfer process between glucose and Ru(dmo–bpy)_2_Cl_2_/GDH/PDA-MWCNT/SPCEs [38]. The electrons from glucose oxidation by GDH–FAD are transferred to the mediator to reduce Ru^3+^ to Ru^2+^. The electrons from reduced Ru(dmo–bpy)_2_Cl_2_ are then moved to the electrode for making catalytic currents.

**Figure 5:**
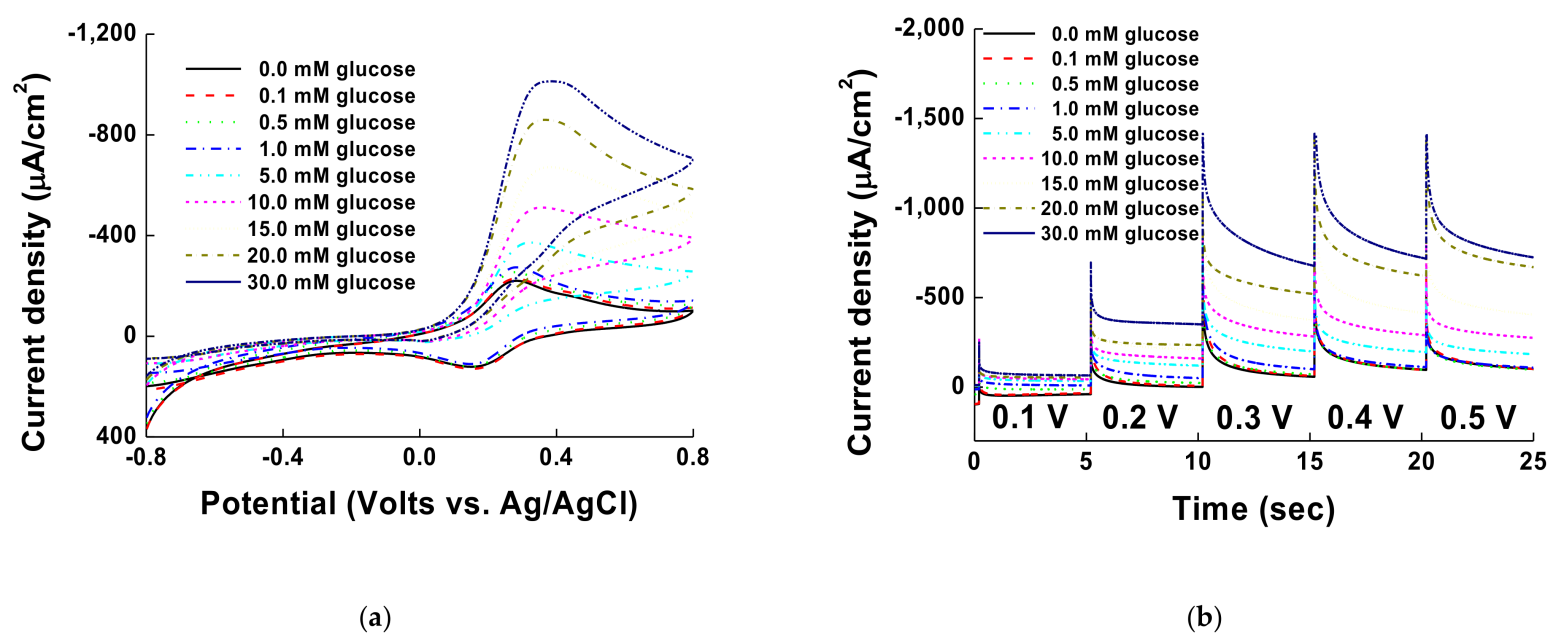

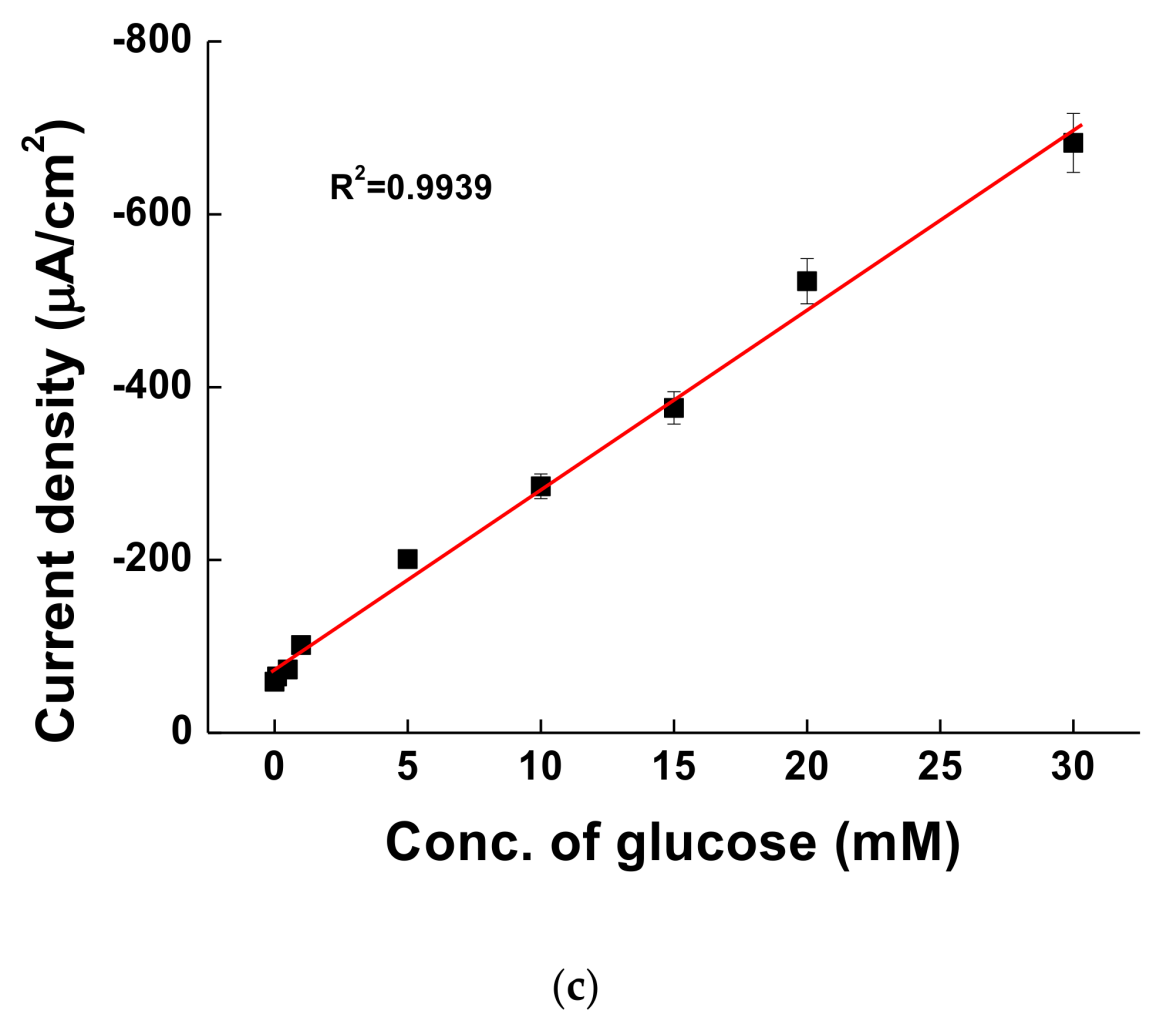
(**a**) Cyclic voltammograms and (**b**) multi-potential step (MPS) results of measuring glucose at concentrations of 0.0, 0.1, 0.5, 1.0, 5.0, 10.0, 15.0, 20.0, and 30.0 mM in PBS (pH 7.0). (**c**) Calibration curve of glucose concentration from 0.0 to 30.0 mM at 0.3 V of MPS in (**b**).

The cyclic voltammograms in Figure 5a show the catalytic currents at glucose concentrations of 0.0, 0.1, 0.5, 1.0, 5.0, 10.0, 15.0, 20.0, and 30.0 mM. The maximum oxidation current was observed at a potential of 0.323 V. In a previous report, Ru(NH_3_)_6_, which was used as a glucose sensor with glucose oxidase enzyme, did not react with GDH. However, Ru(dmo–bpy)_2_Cl_2_ mediator showed excellent reaction with GDH and performance in our PDA-MWCNT-based electrode. Figure 5b shows the MPS results at various glucose concentrations of 0.0, 0.1, 0.5, 1.0, 5.0, 10.0, 15.0, 20.0, and 30.0 mM. The first 0.2 s is for quiet time and afterwards, the voltage was changed every 5 s from 0.1 to 0.5 V. Figure 5c shows the calibration curve at 0.3 V of MPS with linearity (R^2^ = 0.9939) to glucose concentrations. The limit of detection (LOD) was 0.094 mM (*n* = 5) and thus, our system has good performance for quantitative glucose measurements for diagnosis.

### 3.4. Interference Species Testing and Glucose Measurement in Serum

**Figure 6:**
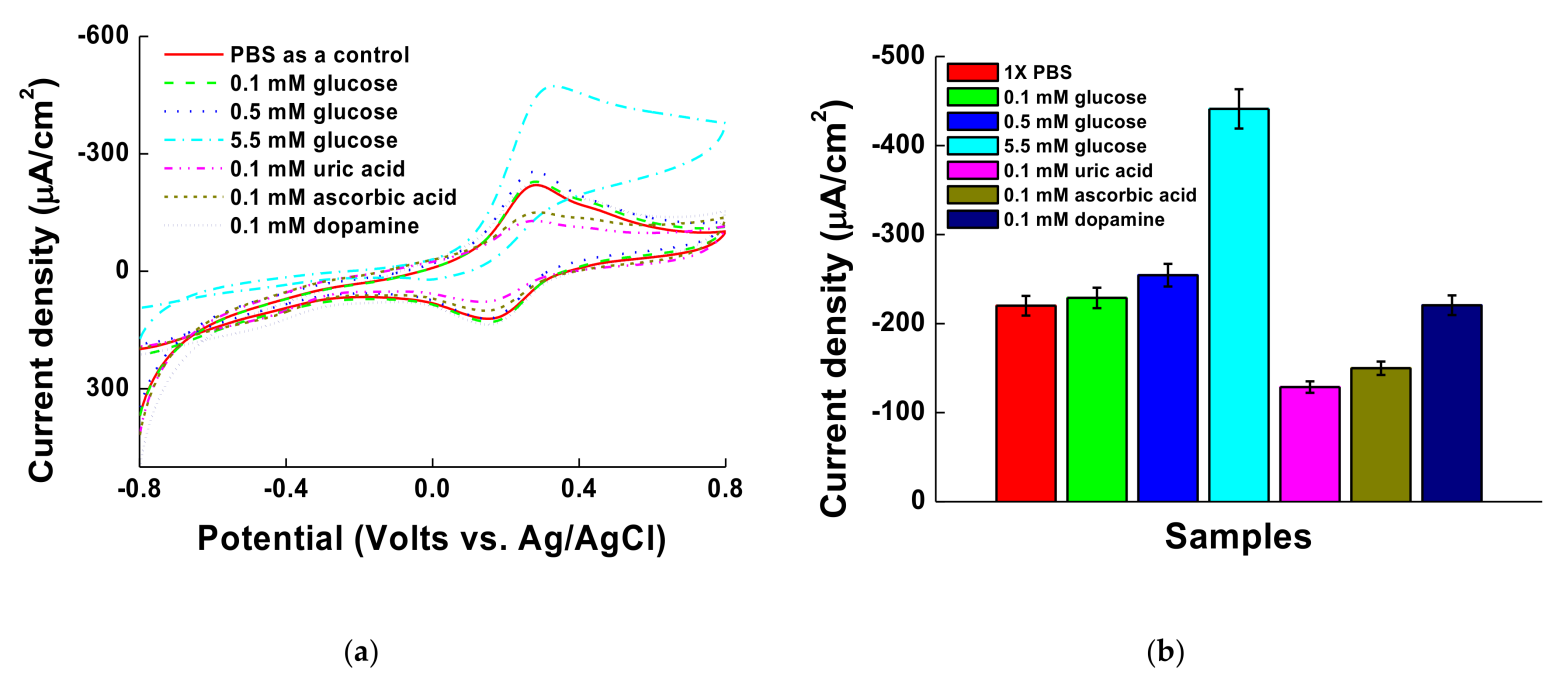
(**a**) Cyclic voltammogram and (**b**) bar graph of 0.1 mM UA, 0.1 mM AA, 0.1 mM DA, and glucose from 0.0 to 5.5 mM with Ru(dmo-bpy)_2_Cl_2_/GDH/PDA-MWCNT/SPCEs (in PBS; pH 7.0).

Figure 6 shows the cyclic voltammograms of measurements for 0.1 mM UA, 0.1 mM AA, 0.1 mM DA, and four concentrations of glucose (0.0, 0.1, 0.5, and 5.5 mM) on the Ru(dmo–bpy)_2_Cl_2_/GDH/PDA-MWCNT/SPCEs. The catalytic currents of glucose (0.1, 0.5, and 5.5 mM) were observed at the 0.3 V potential. The 0.1 mM DA showed the catalytic signal, which, however, was negligible at 0.3 V compared to 0.0 mM glucose, while the currents of 0.1 mM AA and 0.1 mM UA decreased due to the electron repulsion with PDA-MWCNT onto the surface of SPCEs. These results have shown Ru(dmo-bpy)_2_Cl_2_/GDH/PDA-MWCNT/SPCEs can be used for sensing glucose selectively.

**Figure 7:**
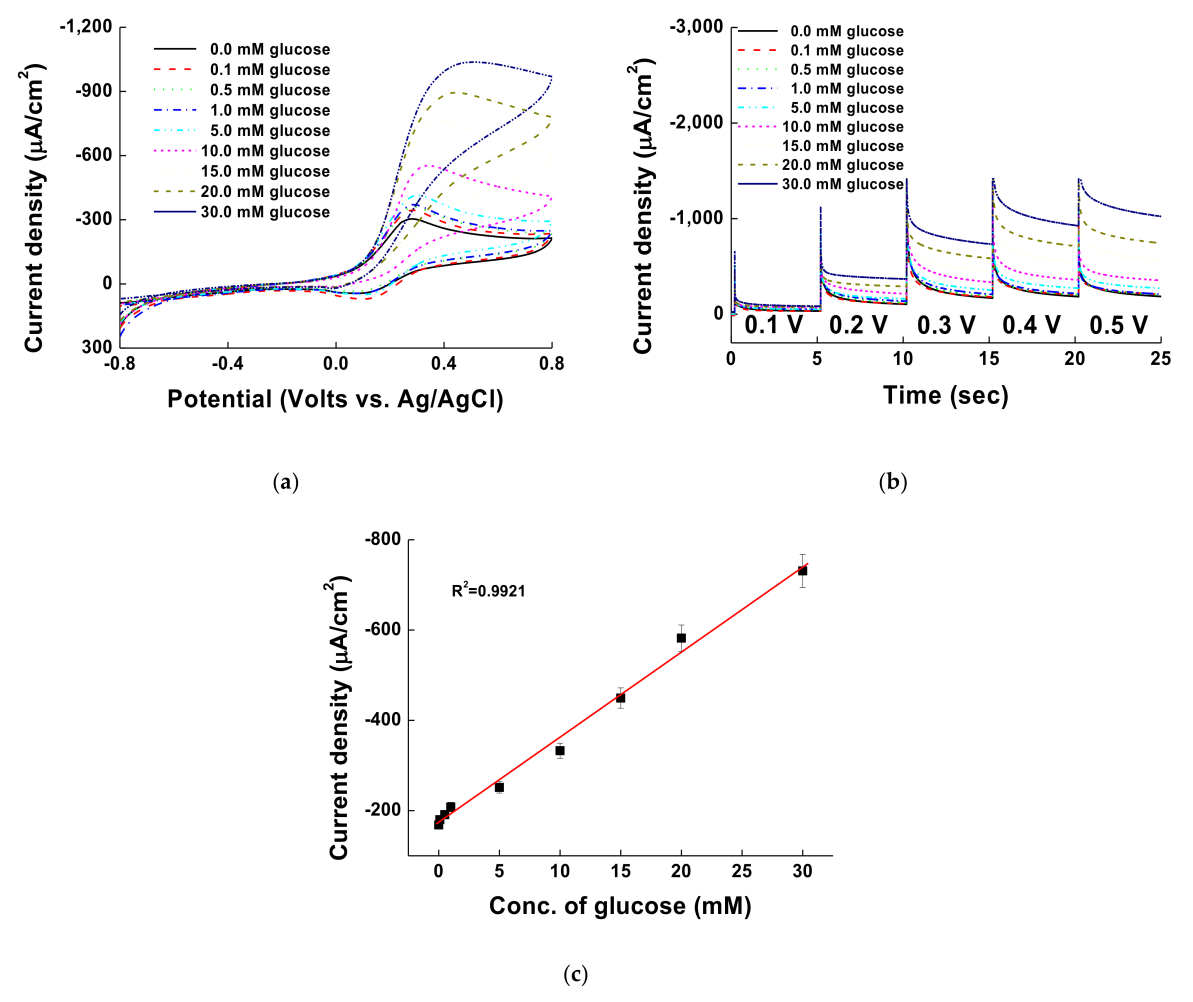
(**a**) Cyclic voltammograms and (**b**) multi-potential step (MPS) results of measuring glucose at concentrations of 0.0, 0.1, 0.5, 1.0, 5.0, 10.0, 15.0, 20.0, and 30.0 mM in serum. (**c**) Calibration curve of glucose concentration from 0.0 to 30.0 mM in serum at 0.3 V of MPS in (**b**).

Additionally, CV and MPS were measured by spiking glucose to human serum samples in the prepared Ru(dmo–bpy)_2_Cl_2_/GDH/PDA-MWCNT/SPCEs. Measurements were carried out by adding 20 μL of serum sample and 20 μL of glucose to a total concentration of 0.0, 0.1, 0.5, 1.0, 5.0, 10.0, 15.0, 20.0, and 30.0 mM. Three serum samples were provided by healthy humans, and experiments were conducted with five sets of electrodes (*n* = 5) to confirm reproducibility. The results of CV and MPS measurements of glucose-spiked serum samples are shown in Figure 7a and b, respectively. CV with high glucose concentration shifted as compared to the results in Figure 5a, and MPS currents at 0.4 and 0.5 V increased as compared to the results in Figure 5b, due to the oxidative species in serum. However, it did not affect the measurement of glucose level.

There was a direct correlation between the current density signals and glucose concentration in serum, which further demonstrates the selectivity in the Ru(dmo-bpy)_2_Cl_2_/GDH/PDA-MWCNT/SPCEs system. Figure 7c shows the calibration curve at 0.3 V of MPS with linearity (R^2^ = 0.9921) to glucose concentrations. The limit of detection (LOD) was 0.584 mM (*n* = 5). Additional intra-assay was performed, and the analysis indicates reproducibility and accuracy of our Ru(dmo–bpy)_2_Cl_2_/GDH/PDA-MWCNT/SPCEs system (Table 2). Additionally, the MWCNT/SPCE-based glucose sensors were compared with the literature in Table 3. Overall, our platform with optimized Ru(dmo-bpy)_2_Cl_2_/GDH/PDA-MWCNT/SPCEs showed excellent performance for sensing the glucose level.

## 4. Conclusions

Glucose sensor using the GDH enzyme is attractive compared to the GOx-based glucose sensor, in that it can measure glucose without the effect of oxygen. A system of enzyme electrodes that transfers electrons generated by the selective oxidation of glucose in GDH to the electrode to confirm the oxidation catalytic current is important in fixing the mediator and enzyme on the electrode surface. In this work, PDA-MWCNT, which is wrapped with PDA to the MWCNT, was adsorbed on the SPCEs and produced a high oxidation catalytic current. We synthesized various proportions of PDA-MWCNT via the ultra-sonication method and found that the PDA-MWCNT weight ratio of efficient transfer was 5.10:1. In addition, Ru(dmo–bpy)_2_Cl_2_ mediator was adsorbed with GDH onto the PDA-MWCNT/SPCEs to transfer the electron successfully. The oxidative catalyst currents produced by glucose were linearly proportional to glucose concentration, showing the good performance of our Ru(dmo–bpy)_2_Cl_2_/GDH/PDA-MWCNT/SPCEs as an electrochemical sensor for the quantification of glucose from 0.0 to 30.0 mM with a LOD of 0.094 mM (*n* = 5) in PBS. The Ru(dmo–bpy)_2_Cl_2_/GDH/PDA-MWCNT/SPCEs was selective for the detection of glucose, and it did not react with the interference species in 0.3 V. Additionally, it showed good results for the quantification of glucose from 0.0 to 10.0 mM with a LOD of 0.584 mM (*n* = 5) in serum. Taken together, we have demonstrated that our system can be used for the development of electrochemical glucose sensors, while its reusability remains to be improved.

## Figures and Tables

**Table 1:** Summary of the ratio between PDA and MWCNT by TGA and HR-TEM.

Composite (Ratio of Amount)	TGA			HR-TEM
	Mass Percentage of PDA (%)	Mass Percentage of MWCNT (%)	Ratio (PDA/MWCNT)	Average of PDA Thickness (nm)
PDA–MWCNT (4:1)	72.9	19.1	3.81:1	4.38 ± 0.22
PDA–MWCNT (5:1)	82.2	16.1	5.10:1	4.87 ± 0.29
PDA–MWCNT (6:1)	82.1	14.6	5.62:1	6.28 ± 0.51
PDA–MWCNT (7:1)	86.0	12.3	6.99:1	8.86 ± 0.48
PDA–MWCNT (8:1)	85.5	11.2	7.63:1	11.68 ± 0.83
PDA–MWCNT (9:1)	83.6	9.6	8.71:1	19.9 ± 1.02

**Table 2:** Determination of glucose onto Ru(dmo–bpy)_2_Cl_2_/GDH/PDA-MWCNT/SPCEs and validation using MPS from the PBS and spiked serum sample (*n* = 3).

Assay	Sample	Nominal Concentration (mM)	Calculated Concentration (mM)	RSD (%)	Accuracy (%)	LOD (mM)
Intra	PBS	0.1	0.101	3.47	101.28 ± 3.468	0.094
		5.0	5.099	4.67	101.99 ± 4.667	
		15.0	15.801	3.56	105.34 ± 3.559	
		30.0	29.574	1.02	98.58 ± 1.018	
	Spiking in Serum	0.1	0.102	4.92	102.16 ± 4.920	0.584
		5.0	5.061	0.89	101.22 ± 0.887	
		15.0	14.73	1.57	98.22 ± 1.569	
		30.0	29.63	1.15	98.77 ± 1.147	

**Table 3:** Comparison with other glucose sensors reports based on MWCNT/SPCE.

Electrode Type	Solution Type	Measurement Technique	Applied Potential(V)	Linear Range(mM)	LOD (mM)	Ref.
GOx/Pt–MWCNTSPCE	0.1 M PBS, pH 7.5	Chronoamperometry	−0.5	0.365–1.446	0.08326	[39]
GOx–SWNT–PVI–Os/SPE	PB, pH 7.4	Chronoamperometry	0.3	0.2–7.5	0.1	[40]
GOx/Pt/rGO/P3ABA/SPCE	PBS, pH 7.4	Chronoamperometry	0.5	0.25–6.0	0.0443	[41]
Ru(dmo–bpy)_2_Cl_2_/GDH/PDA-MWCNT/SPCEs	PBS, pH 7.0	MPS	0.3	0.1–30.0	0.094	Our work Our work
	Serum	MPS	0.3	0.1–30.0	0.584	

## Data Availability

Not applicable.

## Supplementary Materials

The following are available online at https://www.mdpi.com/article/10.3390/membranes11060384/s1, Figure S1: pictures of the (a) dispersed and (b) centrifuged samples, and the (c) supernatant after centrifuging the PDA-MWCNTs., Figure S2: electrochemical system composed of the working, counter, and reference electrodes. Figure S3. TEM image of MWCNTs used in this study.
