# Supplementary material for: Development of a Glucose Sensor Based on Glucose Dehydrogenase Using Polydopamine-Functionalized Nanotubes
Source: Membranes (Basel). 2021 May 24;11(6):384. doi: 10.3390/membranes11060384 (PMC8225004; doi:10.3390/membranes11060384)
Supplement: Supplementary file 1 [file membranes-11-00384-s001.zip › membranes-1209818-supplementary.pdf]

membranes

*Supplementary Material*

# Development of a Glucose Sensor Based on Glucose Dehydrogenase using Polydopamine-Functionalized Nanotubes

**Won-Yong Jeon [1], Hyug-Han Kim [2], Young-Bong Choi [2,*]**

1 School of Chemical Engineering and Biomedical Institute for Convergence at SKKU (BICS), Sungkyunkwan University, Suwon, 16419, Republic of Korea; powerwy@skku.edu (W.-Y.J.)
2 Department of Chemistry, College of Science & Technology, Dankook University, Dandae-ro, Cheonan-si, Chungnam 31116, Republic of Korea; hankim@dankook.ac.kr
* Correspondence: chem0404@dankook.ac.kr (Y.-B.C.); Tel.:+82-41-550-3437, Fax.:+82-41-559-7860.

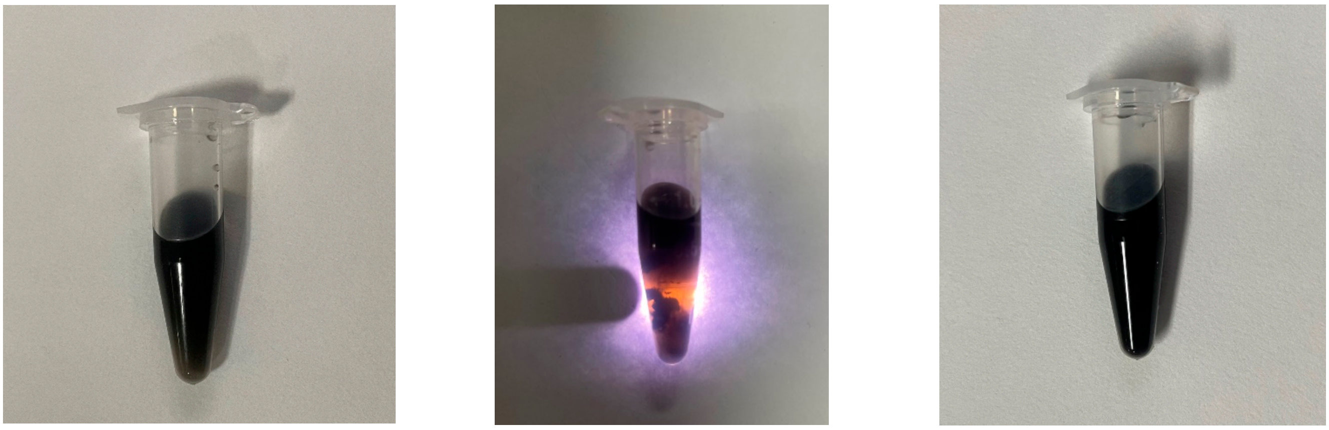

**(a)** **(b)** **(c)**

**Figure S1.** Pictures of the (**a**) dispersed and (**b**) centrifuged samples, and the (**c**) supernatant after centrifuging the PDA-MWCNTs.

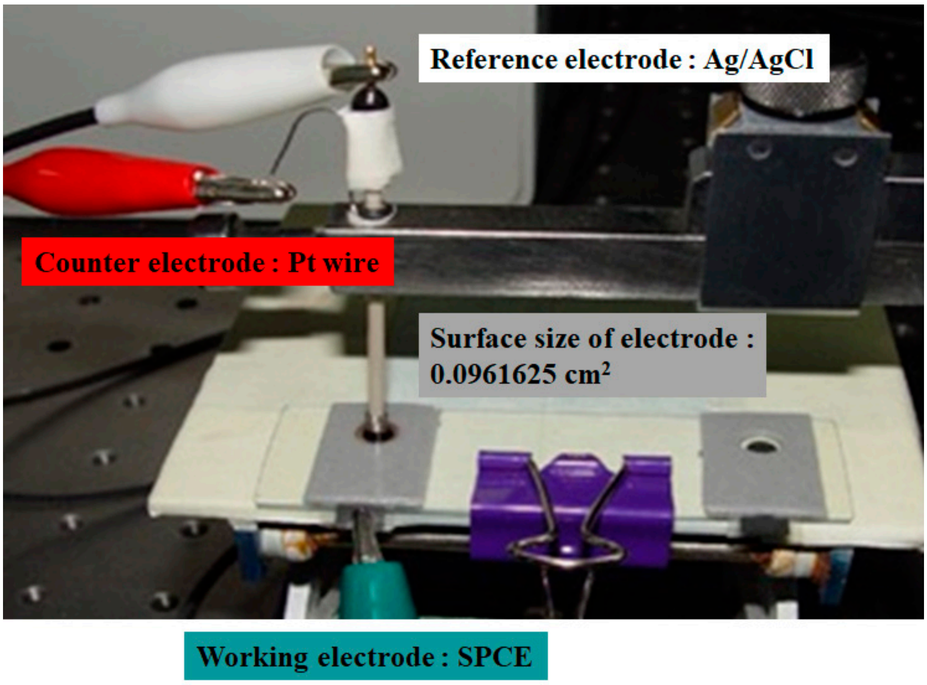


**Figure S2.** Electrochemical system composed of the working, counter, and reference electrodes.

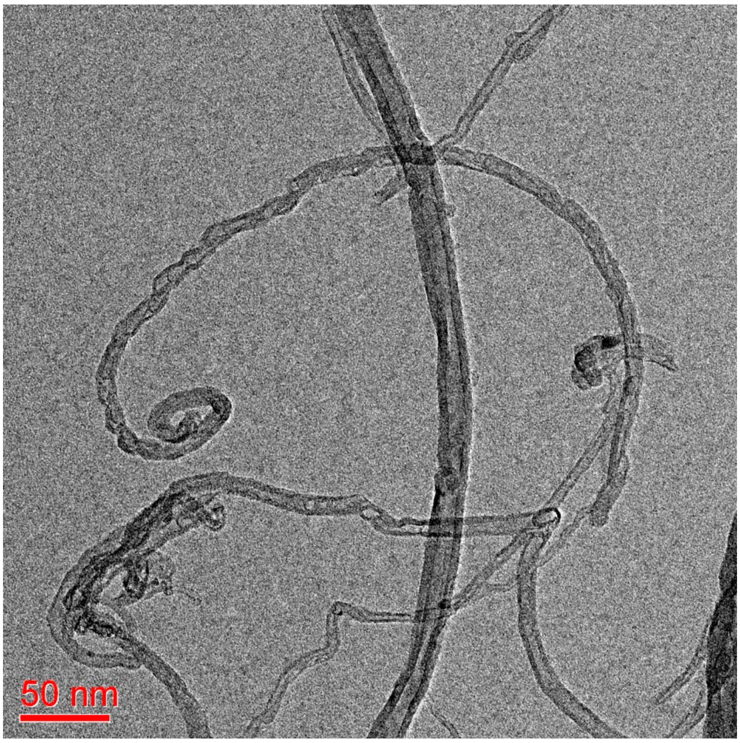


**Figure S3.** TEM image of MWCNTs used in this study.
